# New role for the anandamide metabolite prostaglandin F_2α_ ethanolamide: Rolling preadipocyte proliferation

**DOI:** 10.1016/j.jlr.2023.100444

**Published:** 2023-09-18

**Authors:** Besma Boubertakh, Olivier Courtemanche, David Marsolais, Vincenzo Di Marzo, Cristoforo Silvestri

**Affiliations:** 1 Centre de Recherche de l'Institut Universitaire de Cardiologie et de Pneumologie de Québec (CRIUCPQ), Département de médecine, Faculté de Médecine, Université Laval, Québec, Canada; 2 Institut sur la Nutrition et les Aliments Fonctionnels (INAF), Centre NUTRISS, Université Laval, Québec, Canada; 3 Canada Excellence Research Chair on the Microbiome-Endocannabinoidome Axis in Metabolic Health (CERC-MEND), Université Laval, Québec, Canada; 4 École de Nutrition, Faculté des Sciences de l'Agriculture et de l'Alimentation (FSAA), Université Laval, Québec, Canada

**Keywords:** Lipids, Adipose tissue, Adipocytes, Adipogenesis, 3T3-L1, Cell cycle, MAPKK, Bimatoprost, PPARG, Obesity

## Abstract

White adipose tissue regulation is key to metabolic health, yet still perplexing. The chief endocannabinoid anandamide metabolite, prostaglandin F_2α_ ethanolamide (PGF_2α_EA), inhibits adipogenesis, that is, the formation of mature adipocytes. We observed that adipocyte progenitor cells—preadipocytes—following treatment with PGF_2α_EA yielded larger pellet sizes. Thus, we hypothesized that PGF_2α_EA might augment preadipocyte proliferation. Cell viability MTT and crystal violet assays, cell counting, and 5-bromo-2′-deoxyuridine incorporation in cell proliferation ELISA analyses confirmed our prediction. Additionally, we discovered that PGF_2α_EA promotes cell cycle progression through suppression of the expression of cell cycle inhibitors, p21 and p27, as shown by flow cytometry and qPCR. Enticingly, concentrations of this compound that showed no visible effect on cell proliferation or basal transcriptional activity of peroxisome proliferator-activated receptor gamma could, in contrast, reverse the anti-proliferative and peroxisome proliferator-activated receptor gamma-transcription activating effects of rosiglitazone (Rosi). MTT and luciferase reporter examinations supported this finding. The PGF_2α_EA pharmaceutical analog, bimatoprost, was also investigated and showed very similar effects. Importantly, we suggest the implication of the mitogen-activated protein kinase pathway in these effects, as they were blocked by the selective mitogen-activated protein kinase kinase inhibitor, PD98059. We propose that PGF_2α_EA is a pivotal regulator of white adipose tissue plasticity, acting as a regulator of the preadipocyte pool in adipose tissue.

## Graphical Abstract

**Figure:**
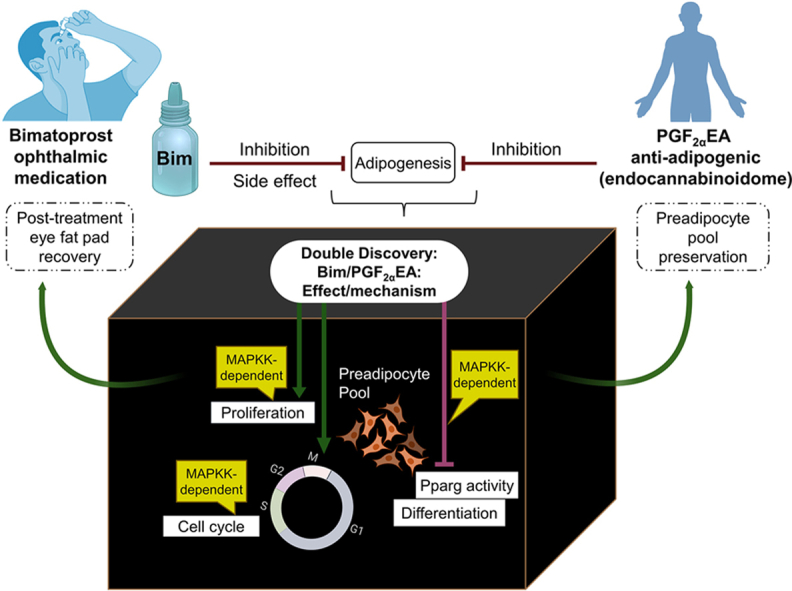

In this digital era, we frequently receive updates on our cell phones, but we lack them on our live cells. This is more critical when it comes to cells of the adipose tissue, particularly the white depots of such tissue (white adipose tissue, WAT), as not only they are located in different parts of the body but they also play several seminal roles (1, 2). The WAT is a dynamically, metabolically, and endocrinologically active tissue (3), with an enormous capacity to expand in size, making it distinct from other tissues (4). While the body contains beige and brown adipose tissue (5), which chiefly play a role in nonshivering thermogenesis, most body fat is stored in the WAT (6), which has a large spectrum of functions ranging from mechanical roles, such as body insulation and supportive cushion for other tissues and organs, as in the case of orbital WAT for the eye, to secretory and energy storage roles (7, 8). Mature adipocytes are the predominant cells of WAT; yet, their precursors—preadipocytes—present primordial roles that are determinants of WAT cellular architecture (9). Importantly, preadipocytes are at the core of WAT organogenesis, mechanical and metabolic preservation, and reactivity (10). However, pivotal regulators of these cells are still largely unknown, and recent papers are continually proposing novel candidates, both eobiotic, such as receptor activator of nuclear factor-κB (11) and fibroblast growth factor 6 (12) and xenobiotic, such as thiazolidinediones like rosiglitazone (Rosi) (13).

Adipogenesis is the differentiation cortege that transforms preadipocytes to mature adipocytes, which then store the body extra energy in the form of triglyceride stocks, in their large vacuole (14, 15). This process is thus required for both physiological fat building and energy storage (16). Adipogenesis, therefore, normally precludes ectopic lipid deposit and subsequent vital organ insulin resistance (17), as it underlies adipocyte hyperplasia at the expense of hypertrophy, which instead constitutes a main attribute of morbid adiposity (4, 18). As mature adipocyte storage capacity is limited, excessive triglyceride accumulation in these cells leads to hypertrophy, which might drive WAT, and subsequently whole body, functional and structural damage, in part through the occurrence of chronic inflammation (17, 18, 19). Thus, adipogenesis is a crucial physiological process for the WAT, for which the organism certainly possesses tight monitoring mechanisms, in which the anti-adipogenic metabolite prostaglandin F_2α_ ethanolamide (PGF_2α_EA)—also named prostamide F_2α_—derived from the enzymatic oxidation of the pro-adipogenic endocannabinoid anandamide has been implicated (20, 21, 22). Yet, PGF_2α_EA, a member of the endocannabinoidome (eCBome) (23), has not been investigated in the context of preadipocyte biology so far.

Anandamide (*N*-arachidonoyl-ethanolamine; AEA) belongs to the endocannabinoid system (eCBS) network of signaling lipids, which presents overarching pathophysiological links with WAT dynamics, functioning, and metabolic networks (24, 25, 26, 27). Factually, relevant research has unveiled valuable clarifying snippets in terms of delineating many diseases pathways, including adipose tissue-related mechanisms regulating energy homeostasis (28). The eCBS mediators are the endocannabinoids, AEA and 2-arachidonoyl-glycerol, which are critical regulators of the WAT and often dysregulated in obesity (23, 28, 29, 30). AEA levels increase during adipogenesis (31), and this eCB is suggested to upregulate this process through activation of cannabinoid receptor 1 and peroxisome proliferator-activated receptor gamma (PPARG) (20, 32, 33). Of momentous interest, certain eCB effects can be mediated—or sometimes opposed—by their bioactive metabolites, thus engendering a wider network of lipid molecules, their target receptors, and metabolizing enzymes, which overlaps with the eCBS pipeline, in a more sophisticated one, known as the eCBome (34, 35). This expanded network of eCB-related signals also includes eCB-like molecules that are not eCB metabolites and—like the eCBS—inextricably crosses obesity and WAT paths (34, 36, 37, 38).

In contrast to arachidonic acid-derived prostaglandin F_2α_, PGF_2α_EA and its synthetic analog bimatoprost (Bim) have been suggested to owe both their intra-ocular pressure-lowering and anti-adipogenic effects to the activation of a heterodimeric receptor consisting of the canonical prostaglandin F_2α_ receptor and an alternately spliced prostaglandin F_2α_ receptor alt4 variant that is still not completely characterized (21, 39, 40, 41, 42, 43, 44, 45). However, like prostaglandin F_2α_ , Bim and PGF_2α_EA inhibit the early adipogenic program, by reducing the *Pparg* and CCAAT enhancer-binding protein alpha (*Cebpa*) gene expression downstream of mitogen-activated protein kinase/extracellular signal-regulated kinases (MAPK/ERK) signaling (21, 46). Interestingly, patients under ophthalmic Bim therapy present quick recovery of eye fat pads after drug cessation (47, 48), suggesting that treatment results in the maintenance of a ready pool of adipocyte precursors that can differentiate and restore the ocular fat pad. Ophthalmology postmarketing clinical surveillance reports indicated that treatment with Bim is associated with a significantly higher load of periorbital adipocytes and lower lipid volume per cell, compared to untreated patients (49). Additionally, a more recent study that injected Bim on rat orbital fat presented similar results (50). However, such observations have barely been interpreted beyond the ocular context (51, 52), although they might strongly relate to WAT and obesity research in as much as this is concerned with fat pads in other districts of the body (53). Indeed, these observations have contributed to paving the way for our hypothesis.

In the present work, we hypothesized that the eCB metabolite PGF_2α_EA induces preadipocyte proliferation. We based this hypothesis on the convergence resulting from our previous findings, current clinical and research knowledge, and our empirical observation that: (i) Bim and PGF_2α_EA inhibit early adipogenesis, and 3T3-L1 preadipocytes produce significant levels of PGF_2α_EA, which decrease dramatically upon the induction of differentiation (21); (ii) patients under ophthalmic Bim therapy witness the reversibility of eye fat pads, after drug cessation (47); (iii) both Bim and PGF_2α_EA show dependence on MAPK signaling for their early anti-adipogenic effects (21), and this signaling pathway is also a well-established driver of cell proliferation (54); (iv) treating preadipocytes with these molecules in our laboratory generated bigger cell pellets, after cellular centrifugation, compared to untreated cells. Accordingly, we hypothesized that PGF_2α_EA and Bim could trigger preadipocyte proliferation, potentially in a MAPK signaling-dependent manner. To verify this hypothesis, we applied a combination of pertinent cell and molecular biology techniques. These inspections provided additional knowledge about WAT physiology and the role of PGF_2α_EA, as well as insights into the potential pertinence of Bim to obesity research. We propose a crucial role of PGF_2α_EA in WAT biology, as a guardian of preadipocyte reserve, by upregulating preadipocyte proliferation and downregulating its differentiation. The potential implication of MAPK kinase (MAPKK; MEK) pathways for both effects was also investigated, both for PGF_2α_EA and Bim. This work might lead to new therapeutic strategies targeting WAT plasticity through favoring hyperplasia to outbalance hypertrophy and hence morbid obesity.

## Materials and Methods

### Cell culture

We utilized the same murine cellular model, that is, 3T3-L1, as our initial study that proved for the first time the anti-adipogenic effect of PGF_2α_EA and the same efficient treatment concentrations, namely 1 μM for Bim and 10 μM for PGF_2α_EA, as well as the same inhibitory tool, which was PD98059 (PD) at 5 μM; a potential and selective MAPK/ERK kinase (MEK) inhibitor (21, 55, 56, 57). We bought PGF_2α_EA and PD from Cayman Chemical Company, while Bim (AGN 192024) was kindly offered by Allergan Pharmaceuticals. Besides 3T3-L1 (a generous gift from Professor Mathieu Laplante; Laval University), we utilized human embryonic kidney (HEK293T) and human hepatoma (HepG2) cells, both kindly offered by Professor André Marette; Laval University. The cells were cultured in DMEM-high glucose (Gibco) containing 10% FBS (Gibco for 3T3-L1 and HyClone for the other two cell lines) at 37°C and 5% CO_2_ in a humidified incubator. Trypsin-EDTA solution for 3T3-L1 and HepG2 passaging was from Sigma, while HEK293T cells were detachable by simple pipetting. 3T3-L1 cells were consistently subcultured before reaching 70% confluence. Adipogenesis was induced two days postconfluence, that is, at day 0 (D_0_), in DMEM supplemented with 10% FBS (Wisent Bioproducts), insulin (1 μg/ml; Gibco), dexamethasone (0.25 μM; Sigma), and 3-isobutyl-1-methylxanthine (IBMX; 0.5 mM; Sigma). Insulin with 1 μg/ml final concentration was prepared by its 1/4000 dilution in differentiation medium from the purchased solution of 4 mg/ml. Dexamethasone powder was first dissolved in ethanol to prepare a 10 mM mother stock solution, which was diluted to a 1 mM stock in PBS, that was then diluted in differentiation medium as for insulin solution. Regarding IBMX powder, we first prepared a KOH solution of 0.25 M (KOH; Sigma) in sterile Milli-Q water. This solution was used to prepare IBMX stock solution (50 mM), which was then diluted by 100X in differentiation medium. The treatment compounds added to the cells were dissolved in DMSO (Sigma) at the ratio of 1:1000 (0.1%), which served then as the vehicle control.

### MTT cell viability assay

3T3-L1 cells were seeded in 96-well tissue culture plates (Thermo Fisher Scientific) at the density of 2 × 10^3^/well. For these microplates, we opted for a liquid volume of 200 μl/well throughout the paper, unless otherwise indicated. The day ensuing cell plating, the growth medium was just renewed (untreated wells), replenished by adding vehicle control DMSO, or treated with Bim/PGF_2α_EA only, PD solely, or with cotreatments (Bim/PGF_2α_EA plus PD). We had, as well, blank control wells, with growth medium but no plated cells. The same concentrations indicated above apply throughout this manuscript unless designated to the contrary. For the assay on HEK293T cells and HepG2 cells, the density was 10^4^ cells/well and 2.5 × 10^4^ cells/well, respectively. Following the initial two days of a four-day treatment, the medium was changed/treatment renewed, for two other days before the investigation. We ordered 3-(4,5-dimethylthiazol-2-yl)-2,5-diphenyltetrazolium bromide (MTT) powder from VWR Life Science. For MTT working solution preparation, we used PBS (Gibco). Thawed aliquots of MTT (5 mg/ml) in PBS were utilized at the ratio of 20 μl–200 μl medium for each well. The plates were stationed in the cell incubator until crystals form in all cell wells. This needed 90 min for 3T3-L1 and half an hour for the other two cell lines. Immediately afterward, wells media were gently and completely discarded, and the formed formazan crystals were dissolved in 200 μl DMSO (Sigma) per well. We orbitally shook the plates, protected from light, at a medium speed for ten minutes. The optical density (OD) was read at the wavelength 570 nm and at 630 nm for background deduction, on BioTek Synergy H1 Hybrid Microplate Reader. The OD percentage to untreated control was calculated according to the following equation: [(treatment OD-average blank OD) ×100/ (untreated control average OD-average blank OD)].

### Cell biomass crystal violet assay

3T3-L1 cells were plated at the compactness of 2000 cells per well in 96-well microplates. The treatments were started 24 h later in growth media containing the appropriate treatment (DMSO vehicle, Bim/PGF_2α_EA, PD, or cotreatments). The treatments were refreshed two days later, and kept for 48 h, to undergo the protocol indicated by the crystal violet assay kit (ab232855; Abcam; Waltham, Massachusetts), where the crystal violet staining is commensurate with the live cellular biomass. The day of reading, media were fully discarded, and the cellular monolayer was gently washed with the kit washing solution, which was completely removed, to allow for a twenty-minute cell staining, as per the kit manual instructions. Then, the staining solution was discarded, and cells were washed four times and then were air-dried as indicated by the kit manufacturer. Each well received, then, 100 μl of solubilization solution from the kit, and the plate was orbitally shaken at a middle speed for 20 min. The final step was the OD measuring at 570 nm on Thermo Scientific Multiskan® Spectrum microplate spectrophotometer.

### Cell counting

Two thousand 3T3-L1 preadipocytes per well were seeded in 96-well tissue culture plates. The cells were untreated or treated with different testing conditions, one day later. The treatments were prepared and scheduled as for MTT and crystal violet assays. After a total of four days of cellular exposure, media/treatments were withdrawn, and the cells were detached with 50 μl trypsin-EDTA to be counted with a hemocytometer. For HEK293T and HepG2 cells, the density was 10^4^ cells/well and 2.5 × 10^4^ cells/well, respectively. For their cell counting, as for 3T3-L1, the cell density, treatments, and the treatment timetable were identical to those of the corresponding MTT assay. HepG2 cells were detached from the well bottoms, following the same procedure as for 3T3-L1 cells, while we simply used pipetting in 50 μl of PBS to detach HEK293T cells for counting.

### BrdU incorporation-based cell proliferation ELISA

3T3-L1 preadipocytes were plated and treated the following day with DMSO, Bim, or PGF_2α_EA for two days as detailed above and then processed using a 5-bromo-2′-deoxyuridine (BrdU) incorporation cell proliferation ELISA kit (ab126556; Abcam; Waltham, Massachusetts). BrdU was added to the cells two hours prior to the end of treatments. We followed the manufacturer’s instructions throughout all the assay steps including cellular fixing and DNA denaturation, all required washing, and the incubation of anti-BrdU monoclonal detector antibody, peroxidase IgG conjugate, peroxidase substrate, and reaction stop solution. The blank consisted of wells with culture medium but no cells, and the assay background control corresponded to the wells plated with cells and treated with the vehicle control (DMSO) but to which the BrdU reagent was not added (No BrdU). Absorbance was read at the wavelength of 450 nm, in addition to 550 nm for subtraction of background noise.

### Flow cytometry assay

We prepared a 3T3-L1 cell suspension at the concentration of 50,860 cells/ml to plate it at the rate of 7 ml per 10-cm diameter dish (Thermo Fisher Scientific). After one day, the cells were either untreated, that is, underwent simple medium renewal, or treated with DMSO, Bim/PGF_2α_EA, PD, or cotreated for 48 h. Next, the cells were trypsinized to be washed twice with 0.5 ml ice-cold PBS (Gibco). Subsequently, ice-cold 70% ethanol (prepared with 100% anhydrous ethanol, Laval University Biobars Scientific Store and UltraPure™ Distilled Water, Invitrogen) was used to fix and delipidize the cells on ice for 30 min, and they were then kept at −20°C until performing flow cytometry. The cells were washed twice with ice-cold PBS, then resuspended in 400 μl PBS. One hundred microliters of this 3T3-L1 suspension was added to 500 μl of propidium iodide (PI)/RNase staining buffer (BD Biosciences). We incubated the tubes for 15 min at room temperature, unexposed to light, before their injection in the flow cytometer (BD LSRFortessa™). The data analysis relied on FlowJo (Tree Star; version 10.8.1). Cell counts were plotted against linear PI fluorescence units, and gates were manually set to obtain frequencies of cells in the G1, S, and G2 phases. The same gates were used for all analyzed samples.

### Caspase activity assay

3T3-L1 cells were plated and treated under the same conditions applied for the flow cytometry assay (see above). The treatments included DMSO, Bim, PGF_2α_EA, and the apoptosis inducer camptothecin (CPT; 1 μM). After two days, the cultured plates were photographed under a VWR® inverted microscope. Cells were then collected by scraping, counted, and then 1.4 × 10^6^ cells were resuspended in chilled lysis buffer and assayed using a caspase assay kit (ab65607; Abcam; Waltham, Massachusetts) following the manufacturer’s instructions. Although the kit we used for checking the effect on apoptosis in preadipocytes is named caspase 9 assay kit, its manual states that it presents cross-reactivity with other initiator caspases, namely caspases 8 and 10, and with the executioner caspases 3 and 6, allowing for a more comprehensive assessment of global caspase activity (58, 59). Caspase activity was measured as fluorescence using BioTek Synergy H1 Hybrid Microplate Reader, at an excitation and emission of 400 nm and 505 nm, respectively.

### Real-time PCR

Two milliliters of 3T3-L1 fibroblasts suspension of 30,000 cells/ml were seeded per well in six-well plates (Thermo Fisher Scientific), to settle for one day before receiving the following treatments: growth medium (untreated), DMSO, Bim/PGF_2α_EA, PD, or cotreatments, for 48 h. Then, we carefully washed the cells with PBS, and we added 500 μl of TRI Reagent® (Sigma) to each well and instantly frozen them at −80°C for later RNA extractions. Total RNA was isolated using Qiagen RNeasy® Plus Mini Kit. Forthwith, RNA was quantified by BioDrop Touch Duo spectrophotometer (Denville Scientific Inc.). The RNA reverse transcription into cDNA relied on Thermo Fisher Scientific Applied Biosystems High-Capacity cDNA Reverse Transcription Kit. The quantitative PCR was carried out on CFX Opus 384 Real-Time PCR System (Bio-Rad) deploying PowerUp™ SYBR® Green Master Mix (Applied Biosystems), with the following timetable: the temperature was initially set at 50°C for two minutes, then increased to 95°C for another two minutes, followed by a total of 40 thermal cycles of 95°C for 15 s followed by a temperature of 60°C for one minute. The relative quantification of gene expression levels was determined according to the normalized ΔΔ quantitation cycle (Cq) parameter, relative to untreated control. Regarding data analysis, we utilized CFX Maestro Software Version 2.0 (Bio-Rad). We present herein (Table 1) the utilized *Mus musculus* primers reverse and forward sequences, predesigned by Integrated DNA Technologies (IDT).

**Table 1:** Primer sequences for quantitative PCR

Gene Name	Abbreviation	Common Name	Reverse Primer (5'-3') Forward Primer (5'-3')
Cyclin-dependent kinase inhibitor 1A	*Cdkn1a*	p21	CTTGTCGCTGTCTTGCACT AATCTGCGCTTGGAGTGATAG
Cyclin-dependent kinase inhibitor 1B	*Cdkn1b*	p27	GAGCAGACGCCCAAGAAG GCAGTGATGTATCTAATAAACAAGGA
Transformation-related protein 53	*Trp53*	p53	CAGGGAGCGCAAAGAGAG CTCCCGGAACATCTCGAAG
Cyclin-dependent kinase 1	*Cdk1*	Cdk1	GACTACAAGAACACCTTTCCCA CGTTTGGCAGGATCATAGACT
Cyclin-dependent kinase 2	*Cdk2*	Cdk2	GCCTGATTATAAGCCAAGTTTCC GTCATAGTGCAGCATTTGCG
Cyclin-dependent kinase 4	*Cdk4*	Cdk4	GGCTGAAATTGGTGTCGGT CTCACGAACTGTGCTGACG
Cyclin-dependent kinase 5	*Cdk5*	Cdk5	CACCGACTGAGGAACAATGG GGTTACACTTCAATAGGTTCTGC
TATA box-binding protein∗	*Tbp*	Tbp	TGTATCTACCGTGAATCTTGGC CCAGAACTGAAAATCAACGCAG
Hypoxanthine guanine phosphoribosyl transferase∗	*Hprt*	Hprt	CCCCAAAATGGTTAAGGTTGC AACAAAGTCTGGCCTGTATCC
Glyceraldehyde-3-phosphate dehydrogenase∗	*Gapdh*	Gapdh	AATGGTGAAGGTCGGTGTG GTGGAGTCATACTGGAACATGTAG

∗ Housekeeping gene for data normalization.

### Luciferase reporter assay

Since HEK293T cell line is a widely adopted cellular transfection model, we used it for our luciferase assay. We plated HEK293T cells at the density of 240,000 cells/ml at the volume of 0.5 ml per well in 24-well plates (Thermo Fisher Scientific). After 24 h, they were transfected with Transporter 5® Transfection Reagent (Polysciences, Inc.) according to the company technical data sheet. Briefly, the medium was changed into 2% FBS medium two hours ahead of transfecting the cells with a total of 0.5 μg DNA, containing 0.1 μg of each of the plasmids of beta (β)-galactosidase, human PPARG2 (hPPARG2), and empty vector, in addition to 0.2 μg of peroxisome proliferator response element (PPRE)-luciferase plasmid. One day later, the cells were untreated or treated with DMSO, PD, Bim, PGF_2α_EA, or Rosi (0.01 μM; powder from EMD Millipore dissolved in DMSO). Additionally, the cells were subject to more than one treatment, where they were treated with Bim or PGF_2α_EA one hour ahead of Rosi (0.01 μM), with or without PD treatment one hour ahead of Bim/PGF_2α_EA. The treatments lasted 24 h, after which the cells were carefully washed with PBS and lysed with 100 μl of luciferase assay lysis buffer prepared in our laboratory. The plates were then frozen at −80°C. To read the corresponding luminescence, the plates were first orbitally shaken at a middle speed for 30 min at room temperature. For the luminescence reading of PPARG activity, 10 μl from each well was transferred to a corresponding well of a 96-well white plate (Costar). The luciferase assay buffer was then injected at a rate of 25 μl/well for 10 s, followed by plate reading using Luminoskan Ascent luminometer (Thermo Electron). The normalized luminescence readings in relative luminescence units were obtained by dividing the luminometer numbers by those of OD values from β-galactosidase yellow product o-nitrophenol read on BioTek Synergy H1 Hybrid Microplate Reader. For this latter measurement, 10 μl from each well of lysed cells was transferred to a matching well of a 96-well clear plate (Corning), to which we added 90 μl of this enzyme substrate 2-nitrophenyl β-D-galactopyranoside (ONPG) solution mixture that we prepared in-house. The plate was incubated at 37°C for 90 min in a 5% CO_2_ humidified incubator, and we quantified the absorbance at 420 nm. For the lysis buffer, it contained the following: Tris base (25 mM), 2 mM of each of DL-DTT, and *trans*-1,2-diaminocyclohexane-*N*,*N*,*N′*,*N′*-tetraacetic acid monohydrate, glycerol (10%) and Triton™ X-100 (1%). The pH was adjusted to 7.8 with o-phosphoric acid (Thermo Fisher Scientific). We specify that all these reagents were from Sigma. The prepared buffer was aliquoted and frozen at −20°C to be thawed at 4°C for usage. With respect to the ONPG solution, it was basically made by dissolving ONPG powder (Sigma) in 0.1 M sodium phosphate solution to obtain the concentration 4 mg/ml. The sodium phosphate solution of a pH of 7.5 was prepared in the laboratory as well, by mixing 20.5 ml of Na_2_HPO_4_·2H_2_O (0.2 M) with 4.5 ml of NaH_2_PO_4_·2H_2_O (0.2 M), to obtain a solution to which we added 25 ml of Milli-Q water. The 90 μl-per-well-to-add ONPG solution mixture was then made of 22 μl of ONPG solution, 67 μl of 0.1 M sodium phosphate solution, and 1 μl of MgCl_2_ (100X solution). We prepared this latter ingredient with MgCl_2_ (0.2 M; Sigma) and 2-mercaptoethanol (4.5 M; MP Biomedicals) in Milli-Q water. The luciferase assay buffer also consisted of Milli-Q water but that contained 470 μM of the substrate for the luciferase enzyme, that is, D-luciferin (Sigma), besides ingredients that allow for optimal enzymatic activity. Thus, it comprised tricine (20 mM from a 0.5 M stock solution in Milli-Q water), MgSO_4_ (2.67 mM from a purchased 1 M solution), and EDTA (Thermo Fisher Scientific; 0.1 mM from a stock solution of 0.5 M dissolved in Milli-Q water by gradual addition of drops of NaOH [Sigma; 10 M in the same water] while magnetically stirred, resulting in a final pH of 7.8). The list of ingredients for optimum enzymatic activity also contained (MgCO_3_)_4_·Mg(OH)_2_·5H_2_O (1.07 mM), DTT (33.3 mM), coenzyme A sodium salt hydrate (270 μM), and adenosine 5*′*-triphosphate (530 μM). All these reagents were purchased from Sigma; in the form of solid powder, except for MgSO_4_ that was bought in the ready-to-use liquid form. Regarding the plasmids used in this assay, they were kindly offered by Professor André Marette. Their constructs maps can be consulted on Addgene website: PPRE-luciferase (PPRE X3-TK-luc; plasmid #1015); hPPARG2 (plasmid #11439), and on NovoPro Bioscience Inc. website for the empty vector pcDNA3.1+/N-Myc (#V011314). For hPPARG2, the plasmid construct pcDNA3.1-N-Myc-hPPARG2 was produced through molecular cloning techniques. The hPPARG2 gene fragment was first amplified from the pBabe bleo hPPARG2 (Addgene; plasmid #11439) using PCR. The resulting PCR product was then enzymatically digested using KpnI and XbaI restriction enzymes and subsequently inserted into the pcDNA3.1-Myc plasmid vector, which had also been predigested with KpnI and XbaI. This process resulted in the generation of the pcDNA3.1-N-Myc-hPPARG2 plasmid construct. Regarding β-galactosidase plasmid (pCMVbeta), it was a kind offer from Professor Liliana Attisano (University of Toronto), and the plasmid map can be consulted at Addgene website (ATCC: 77177; Clontech PT2004-5).

### Statistical analyses

The experiments described throughout this paper were all repeated at least three times. The central tendency measure presented in the figures is the mean, and the data variability around it was exhibited as the SD. We conducted the statistical ANOVA (one-way) and Šidák’s multiple comparisons post hoc test, with GraphPad Prism Version 9.5.1 (733) Software. The null hypothesis was rejected for *P* values that are less than 0.05.

## Results

### Bimatoprost and PGF_2α_EA increase 3T3-L1 preadipocyte viability

**Fig. 1:**
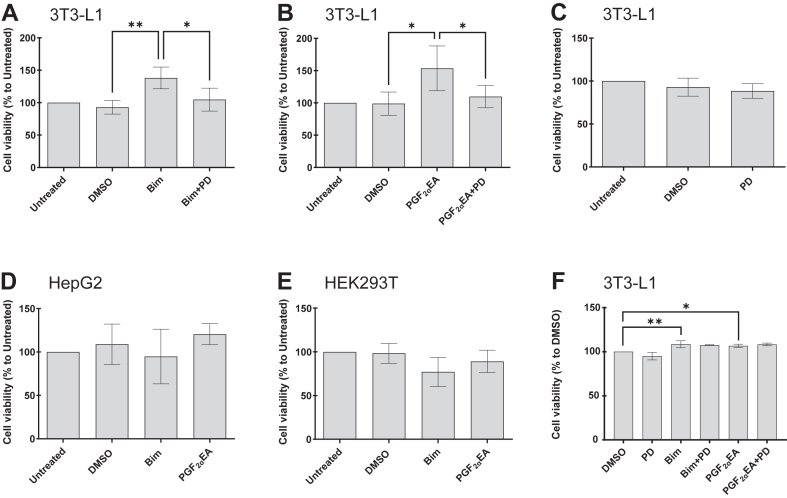
Effect of bimatoprost (Bim) and prostaglandin F_2α_ ethanolamide (PGF_2α_EA) on 3T3-L1 preadipocyte viability. 3T3-L1 preadipocytes (A–C) were untreated or treated with vehicle DMSO (0.1%) or PD98059 (PD; 5 μM) or Bim (1 μM)/PGF_2α_EA (10 μM) without or with PD, for four days. The cell viability was estimated using the MTT assay. HepG2 (D) and HEK293T (E) cells were also untreated or treated with DMSO, Bim, or PGF_2α_EA for MTT assay after four days of treatment. The observed effects on cell viability in 3T3-L1 were further studied using the crystal violet cell viability assay (F) under the same treatment timetable. The results are expressed as percentages relative to the untreated control (MTT assay) or to vehicle control (crystal violet assay) and are illustrated as the mean ± SD, for n = 4 and n = 3 independent bioreplicates, respectively. ∗*P* < 0.05, ∗∗*P* < 0.01. MTT, 3-(4,5-dimethylthiazol-2-yl)-2,5-diphenyltetrazolium bromide.

We initially assessed whether 3T3-L1 preadipocytes exposure to Bim and PGF_2α_EA could show significant effect as determined by MTT assay, which is an indirect measure of cellular viability (proliferation), through measuring cellular metabolic activity (60, 61). The treatment of 3T3-L1 preadipocytes with either Bim or PGF_2α_EA for four days significantly increased cellular viability, compared to vehicle control (DMSO) (Fig. 1A, B). As the anti-adipogenic activity of Bim and PGF_2α_EA is dependent on MAPKK (21), we tested if the effects observed here were also MAPKK-dependent by co-incubating cells with the compounds and MAPKK-selective inhibitor, PD (Fig. 1A, B). The PD treatment per se did not affect cellular viability (Fig. 1C). However, it completely reversed the effects of Bim and PGF_2α_EA in the MTT assay (Fig. 1A, B). We further wanted to explore whether the effect of these prostanoids could be observed on other cell lines. The MTT assay did not indicate any effect of either compound on the cellular viability of the hepatocellular HepG2 (Fig. 1D) nor embryonic kidney HEK293T (Fig. 1E) cell lines, compared to the strong effect on preadipocytes. To further confirm the potential effect of our treatments on 3T3-L1 cell viability, we used the crystal violet cell viability assay kit, which directly quantifies the cellular biomass of the cell monolayer once stained and washed. The treatments were scheduled as for the MTT assay, and the results were consistent with those of this test, as both Bim and PGF_2α_EA increased the cell viability, although PD did not significantly reverse the effect (Fig. 1F).

### Bimatoprost and PGF_2α_EA increase 3T3-L1 preadipocyte proliferation

**Fig. 2:**
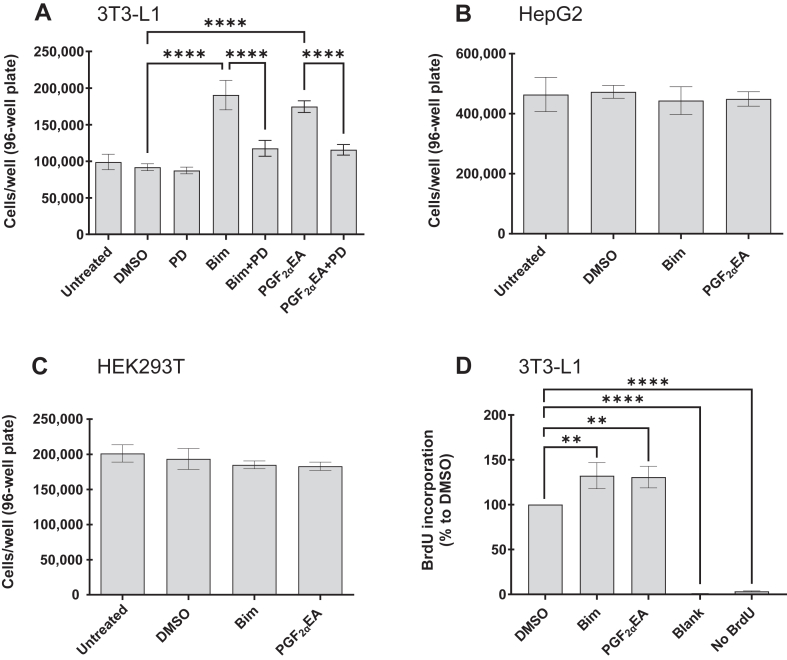
Effect of bimatoprost (Bim) and prostaglandin F_2α_ ethanolamide (PGF_2α_EA) on 3T3-L1 preadipocyte cell number. 3T3-L1 cells were untreated or treated with DMSO (0.1%) or Bim (1 μM)/PGF_2α_EA (10 μM), without or with PD98059 (PD; 5 μM), or PD, for four days, and then counted with a hemocytometer (A). HepG2 (B) and HEK293T (C) cells were also untreated or treated with DMSO, Bim, or PGF_2α_EA, for four days and counted as for 3T3-L1. In addition, a BrdU incorporation cell proliferation ELISA was performed on 3T3-L1 cells that were plated and treated as indicated in (A), with DMSO, Bim, or PGF_2α_EA besides the assay blank (no cells) and background control (cells treated with DMSO, but without BrdU addition; No BrdU), for two days. The colorimetrically measured average BrdU incorporation percentages to DMSO control are presented (D). The results are shown as the mean ± SD of three independent bioreplicates. ∗∗*P* < 0.01, ∗∗∗∗*P* < 0.0001. BrdU, 5-bromo-2′-deoxyuridine.

The data from the MTT and crystal violet assays suggested a potential augmenting effect on 3T3-L1 preadipocyte cell number. Indeed, we had originally observed that the Bim- and PGF_2α_EA-treated cultures resulted in larger cell pellets. Thus, we followed the same treatment schedule described above, and we counted the cells. Both compounds resulted in a strongly statistically significant (*P* < 0.0001) increase in the number of preadipocytes per well (Fig. 2A). In addition, PD treatment strongly reversed the effect of both Bim and PGF_2α_EA (*P* < 0.0001). In contrast, Bim and PGF_2α_EA did not affect cellular proliferation for the other tested cell lines, namely, HepG2 (Fig. 2B) and HEK293T (Fig. 2C). Further investigation measuring BrdU incorporation into proliferating cells corroborated the results of the previous assays, showing that the examined compounds increased preadipocyte proliferation. Indeed, the OD values from both Bim and PGF_2α_EA treatments, which quantify the incorporation of the thymidine analog BrdU into newly synthesized DNA, were significantly higher than the vehicle control (*P* < 0.01 for both) (Fig. 2D). On the other hand, the negligible signals from the blank and background (No BrdU) controls (Fig. 2D) indicated the specificity of BrdU incorporation.

### Bimatoprost and PGF_2α_EA advance 3T3-L1 preadipocytes through the cell cycle phases

**Fig. 3:**
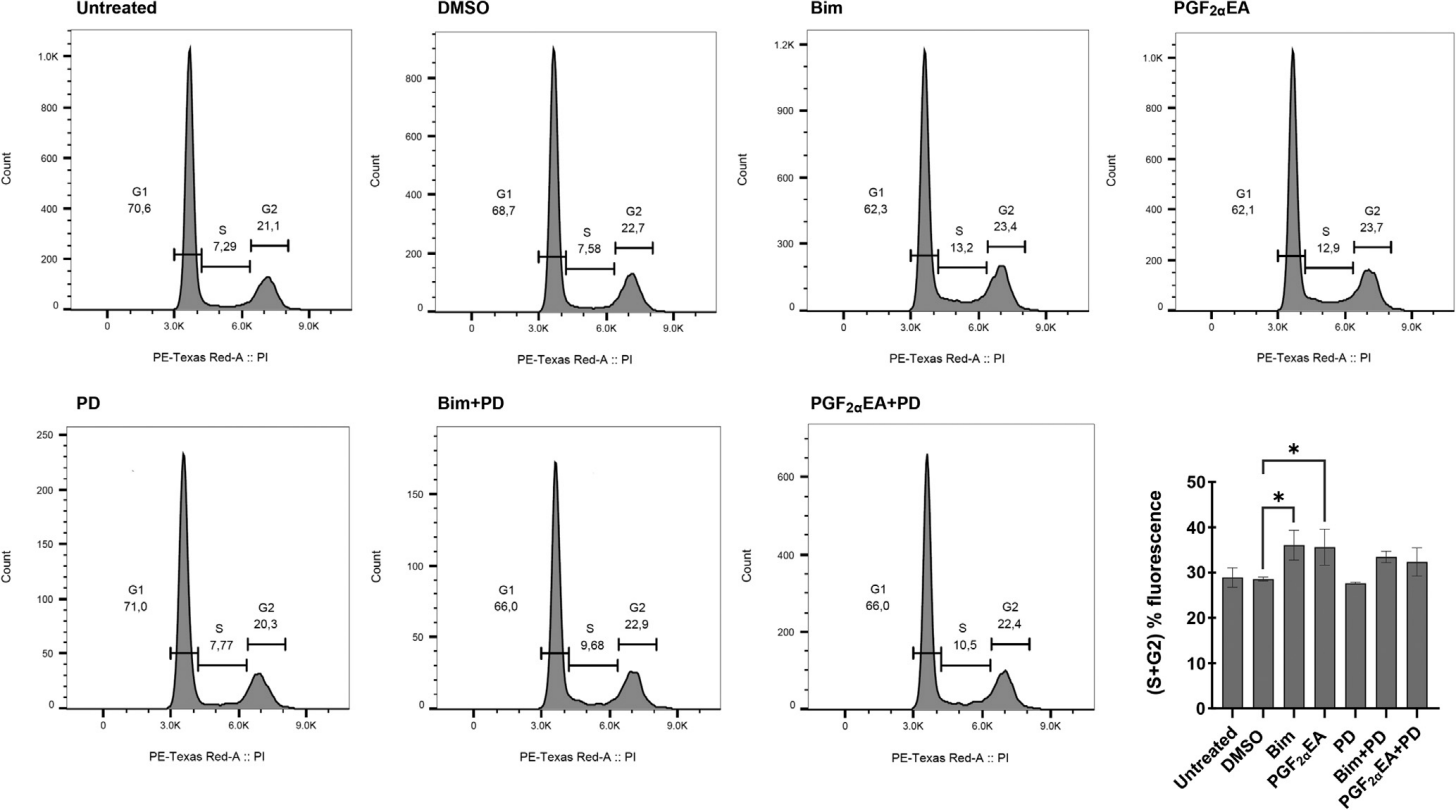
Effect of bimatoprost (Bim) and prostaglandin F_2α_ ethanolamide (PGF_2α_EA) on 3T3-L1 preadipocyte cell cycle. 3T3-L1 cells were either untreated or treated with DMSO (0.1%) or PD98059 (PD; 5 μM) or with Bim (1 μM)/ PGF_2α_EA (10 μM), without or with PD, for two days. The percentage of cells in the G1, S, and G2 cell cycle phases was evaluated by flow cytometry. A representative image of flow cytometry histograms for all treatments is shown in Fig. 3, along with quantification of the cell percentage within the sum of the S and G2 cell cycle phases (Fig. 3 bar chart). The results are shown as the mean ± SD of three independent bioreplicates. ∗*P* < 0.05.

**Fig. 4:**
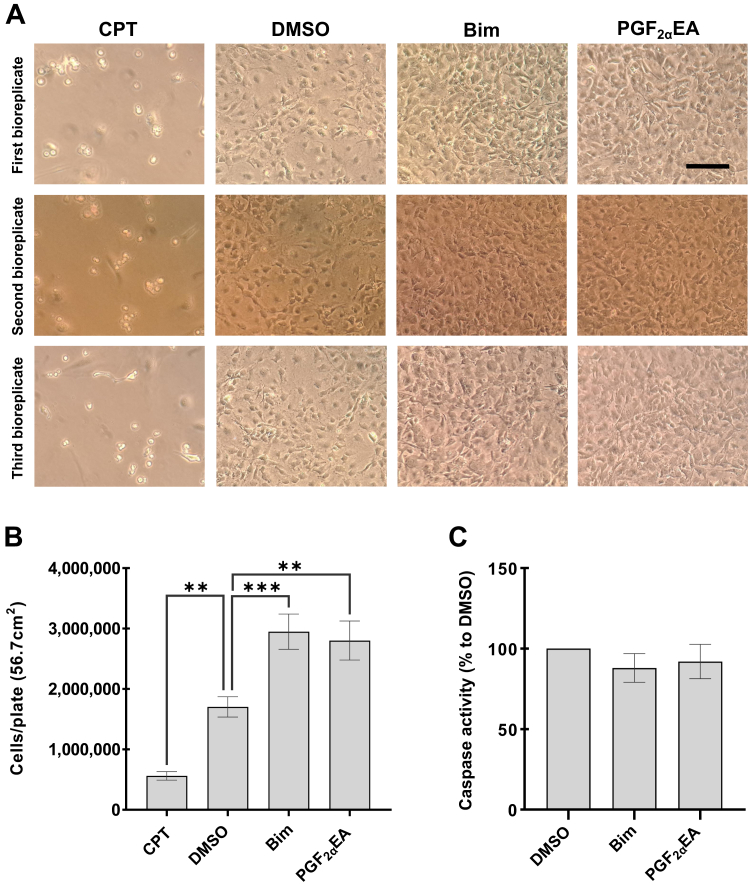
Effect of bimatoprost (Bim) and prostaglandin F_2α_ ethanolamide (PGF_2α_EA) on 3T3-L1 preadipocytes as per microscopic observation and caspase activity assay. 3T3-L1 cells were treated with DMSO (0.1%), Bim (1 μM), PGF_2α_EA (10 μM), or camptothecin (CPT; 1 μM) as a positive control for apoptosis, for two days, after which the cells were photographed under the setting of 10X magnification of an inverted microscope. Representative images of treated cells from each of the three bioreplicates are illustrated (A). Scale bar indicates 75 μm. The total number of cells per plate, as counted with a hemocytometer, was determined (B). Caspase activity, relative to DMSO vehicle control, was determined for both Bim and PGF_2α_EA (C). The results are shown as the mean ± SD of three independent bioreplicates. ∗∗*P* < 0.01, ∗∗∗*P* < 0.001.

To determine whether Bim and PGF_2α_EA altered the 3T3-L1 cell numbers within different phases of the cell cycle, we exposed the cells to our two treatments without or with the MEK inhibitor, besides this inhibitor, vehicle control, and the untreated condition. These treatments were applied at the same concentrations indicated above, for two days. Subsequently, we analyzed the DNA content via flow cytometry. Representative DNA fluorescence histograms from PI/RNase-stained preadipocytes from each treatment condition are shown in Fig. 3. Assessment of the percentage of cells within the sum of the S and G2 cell cycle phases confirmed that both Bim and PGF_2α_EA significantly increased the number of cells that had undergone DNA replication (Fig. 3 bar chart). Importantly, further comparing cotreatments with vehicle DMSO showed that PD co-administration at a per se ineffective dose rendered the effects of Bim and PGF_2α_EA no longer statistically significant. Additionally, the cells were plated and treated under the same conditions to investigate the possibility that these compounds could affect levels of apoptosis in 3T3-L1 cells, by measuring caspase activity, a key driver of apoptosis (62). The cells were photographed and counted at the end of the treatments. CPT-treated cells (to induce apoptosis) displayed apoptotic features that included cell shrinkage, rounding, and detachment (63) (Fig. 4A), and counting these cells revealed significantly reduced cell numbers (Fig. 4A, B). In contrast, cells treated with the vehicle control, DMSO, did not exhibit a similar phenotype (Fig. 4A). Likewise, Bim- and PGF_2α_EA-treated cells did not present apoptotic features but, instead, showed an increase in cell number, which was confirmed through cell counting (Fig. 4A, B). Quantification of caspase activity (the kit utilized is able to assess caspases 3, 6, 8, 9, and 10) showed no effect of either Bim or PGF_2α_EA (Fig. 4C), indicating that these compounds likely do not result in increased 3T3-L1 cell number by decreasing levels of apoptosis. The kit used for caspase activity evaluation was validated with CPT as a positive control in a separate experiment, as per the manufacturer’s instructions (data not shown).

### Bimatoprost and PGF_2α_EA affect the gene expression of 3T3-L1 preadipocyte CDKs and CDK inhibitors

**Fig. 5:**
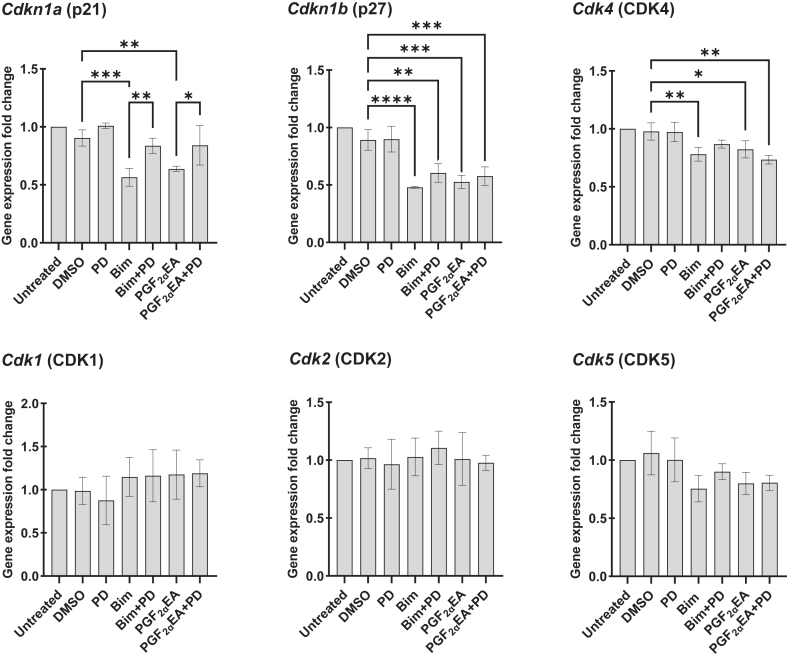
Effect of bimatoprost (Bim) and prostaglandin F_2α_ ethanolamide (PGF_2α_EA) on 3T3-L1 cell cycle cyclin-dependent kinase (CDK) and CDK inhibitor (CDKI) gene expression. 3T3-L1 cells were untreated or treated with DMSO (0.1%), PD98059 (PD; 5 μM), or Bim (1 μM)/ PGF_2α_EA (10 μM), without or with PD, for two days, and then the gene expression of the CDKIs *Cdkn1a*, *Cdkn1b*, and CDKs *Cdk1*, *2*, *4*, and *5* was quantified by quantitative PCR. The results are shown as the mean ± SD of relative gene expression to the untreated control, for three independent bioreplicates. ∗*P* < 0.05, ∗∗*P* < 0.01, ∗∗∗*P* < 0.001, ∗∗∗∗*P* < 0.0001.

For further mechanistic examination of the proproliferative effect of Bim and PGF_2α_EA, we proceeded to analyze the expression of several cyclin-dependent kinase (CDK) and CDK inhibitor (CDKI) genes. Cyclins control the cell cycle advancement through activation of CDKs, which are necessary for proper cell cycle progress and are inhibited by CDKIs (64, 65). Herein, we explored if Bim and PGF_2α_EA affect the gene expression of the CDK 1, 2, 4, and 5 (*Cdk1*, *2*, *4*, and *5*, respectively) and the CDKIs (p21; *Cdkn1a* and p27; *Cdkn1b*) (Fig. 5), besides p53 (*Trp53*; data not shown). The treatment plan corresponded to that followed for the flow cytometry analysis of our drug effects on the cell cycle, that is, for two days. The results indicated that both Bim and PGF_2α_EA strongly downregulated the expression of the genes *Cdkn1a* and *Cdkn1b* (*P* < 0.01) that negatively regulate the progression from G1 to S. For the former, the effect of both treatments was substantially reversed by PD co-exposure. However, PD cotreatment did not reverse the reducing effect of Bim and PGF_2α_EA on *Cdkn1b*, and this was true also for *Cdk4* and for *Cdk5*. We further compared the cotreatment effect to that of vehicle control for these genes to assess whether cotreatment with PD presented a weaker effect than Bim or PGF_2α_EA alone. As expected for p27, the cotreatment with PD rendered Bim effect less statistically significant. This pattern was also observed for PGF_2α_EA, though not statistically considerable. For *Cdk4*, co-exposure to PD rendered the reducing effect of Bim, but not PGF_2α_EA, on gene expression no longer statistically significant. Regarding *Cdk1*, *2*, and *5*, no significant effects were observed.

### The inducibility of bimatoprost and PGF_2α_EA effects

#### Effect of bimatoprost and PGF_2α_EA on PPARG activity

**Fig. 6:**
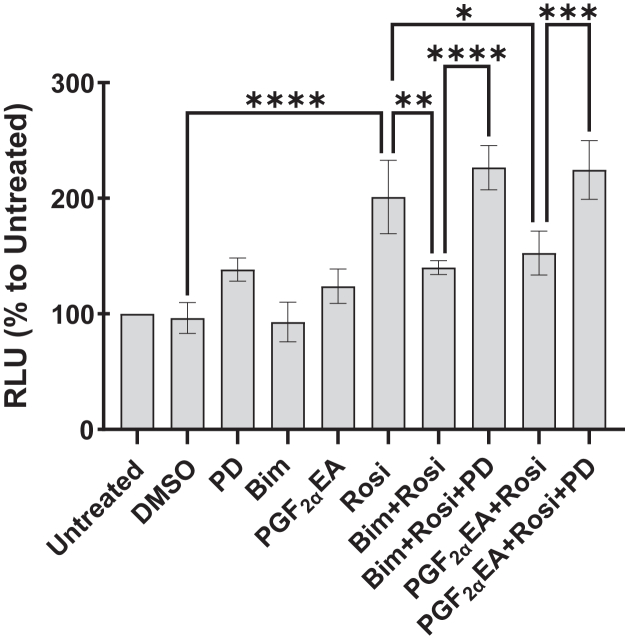
Effect of bimatoprost (Bim) and prostaglandin F_2α_ ethanolamide (PGF_2α_EA) on peroxisome proliferator-activated receptor gamma (PPARG) activity. HEK293T cells were transfected with a PPARG expression construct, the peroxisome proliferator response element (PPRE)-luciferase reporter construct, and constitutively active β-galactosidase plasmid construct. Then, one day posttransfection, the cells were untreated or treated with DMSO vehicle (0.1%) or PD98059 (PD; 5 μM), or Bim (1 μM)/PGF_2α_EA (10 μM) without or with PD, followed by rosiglitazone (Rosi; 0.01 μM) treatment. After 24 h, the luminescence was read and corrected to β-galactosidase activity. The results are depicted as the mean ± SD of relative luminescence units (RLU) presented as percentages to the untreated control, for three independent bioreplicates. ∗*P* < 0.05, ∗∗*P* < 0.01, ∗∗∗*P* < 0.001, ∗∗∗∗*P* < 0.0001.

Previously, we showed that both Bim and PGF_2α_EA inhibit *Pparg* gene expression and adipogenesis (21), in a manner that was sensitive to MAPKK inhibition by PD. However, it is still unknown whether the two molecules affect PPARG activity. To check this potential effect, we utilized a PPARG-dependent transcriptional activity luciferase reporter assay in transiently transfected HEK293T cells. The treatments consisted of vehicle control (DMSO 0.1%), PD (5 μM), Bim (1 μM), or PGF_2α_EA (10 μM), besides the untreated condition. In addition, the cells were exposed to the PPARG agonist Rosi (0.01 μM) alone or an hour after cell incubation with Bim or PGF_2α_EA, to allow for any effects of these prostanoids to occur before adding Rosi. To assess whether any reversing effects of PPARG activation, by Bim/PGF_2α_EA, could be MEK-dependent, the experiment included cell exposure to PD an hour ahead of Bim/PGF_2α_EA treatments that preceded Rosi addition. As shown in Fig. 6, both Bim and PGF_2α_EA substantially lowered Rosi-induced PPARG activity. Importantly, and in accordance with the aforementioned effects of these drugs, the reversal of PPARG activity was completely suppressed by PD treatment. Neither Bim nor PGF_2α_EA affected basal PPARG activity.

#### Effect of bimatoprost and PGF_2α_EA on rosiglitazone inhibition of 3T3-L1 cell proliferation

**Fig. 7:**
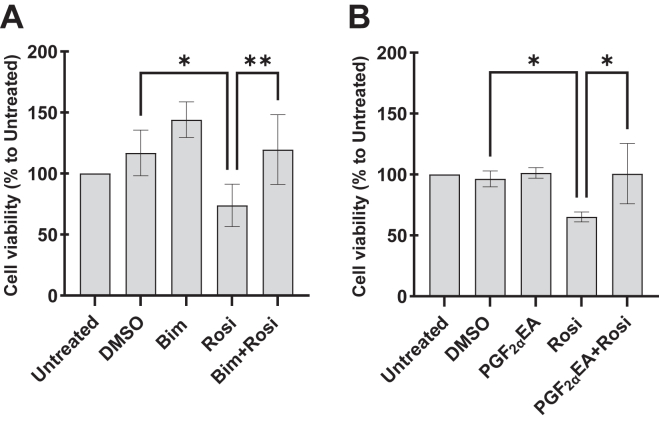
Effect of bimatoprost (Bim) and prostaglandin F_2α_ ethanolamide (PGF_2α_EA) on the inhibition of 3T3-L1 cell proliferation by rosiglitazone (Rosi). 3T3-L1 preadipocytes were untreated or treated with DMSO (0.1%), Bim (0.05 μM; A)/PGF_2α_EA (0.05 μM; B), or Rosi (0.5 μM) without or with Bim or PGF_2α_EA, for four days. Cell viability was quantified with the MTT assay. The results are presented as percentages relative to the untreated control and are shown as the mean ± SD of four (A) or three (B) independent bioreplicates. ∗*P* < 0.05, ∗∗*P* < 0.01. MTT, 3-(4,5-dimethylthiazol-2-yl)-2,5-diphenyltetrazolium bromide.

Albeit PPARG is more widely known as playing a role in adipogenesis, it is also a negative regulator of preadipocyte proliferation as shown for certain thiazolidinediones or their derivatives (66, 67, 68, 69). We exposed 3T3-L1 cells to Bim/PGF_2α_EA at the concentration 0.05 μM, which did not significantly induce cell proliferation, besides the untreated condition, vehicle control DMSO (0.1%), and Rosi at 0.5 μM, the latter of which significantly impeded cellular multiplication and the concomitant treatment consisting of either Bim (Fig. 7A) or PGF_2α_EA (Fig. 7B) with Rosi. This experimental design, which was the same as the aforementioned MTT experiments, aimed at evaluating Bim/PGF_2α_EA capability to reverse Rosi inhibition of cellular proliferation. Indeed, both Bim and PGF_2α_EA, at a concentration that did not induce cell proliferation per se, could reverse the inhibitory effect of Rosi on cell viability (Fig. 7).

#### Effect of bimatoprost and PGF_2α_EA on 3T3-L1 preadipocytes undergoing differentiation

**Fig. 8:**
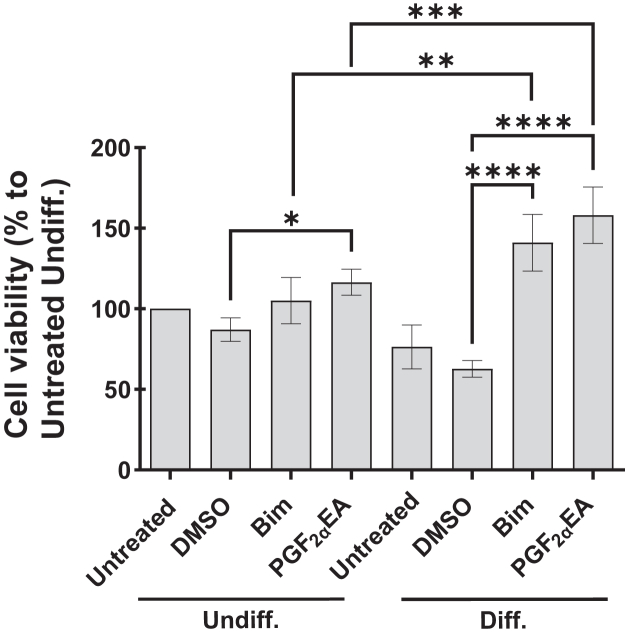
Effect of bimatoprost (Bim) and prostaglandin F_2α_ ethanolamide (PGF_2α_EA) on differentiating 3T3-L1 cells. 3T3-L1 preadipocytes were induced to differentiate (Diff.) or not (Undiff.). In both cases, the cells were untreated or treated with either DMSO (0.1%), Bim (1 μM), or PGF_2α_EA (10 μM), for two days. The cell viability was then quantified with the MTT assay. The results are presented as percentages relative to the untreated undifferentiated control and are illustrated as the mean ± SD of four independent bioreplicates. ∗*P* < 0.05, ∗∗*P* < 0.01, ∗∗∗*P* < 0.001, ∗∗∗∗*P* < 0.0001. MTT, 3-(4,5-dimethylthiazol-2-yl)-2,5-diphenyltetrazolium bromide.

We showed above that both Bim and PGF_2α_EA raise the cell viability of nonconfluent 3T3-L1 preadipocytes. We further examined their effect on preadipocyte viability under the condition of challenged preadipocyte pool through triggering its differentiation. Thus, two days postconfluence, 3T3-L1 cells were untreated or treated for two days with Bim (1 μM) or PGF_2α_EA (10 μM), besides the vehicle control DMSO. These treatments were applied both to undifferentiated (Undiff.) cells and to cells where we triggered the differentiation (Diff.). We observed that Undiff. cell exposure to these prostanoids induced a slight increase of cellular viability, despite the fact that treatments occurred two days postconfluence (Fig. 8 Undiff. section). In contrast, both compounds more than doubled the cell viability compared to vehicle (DMSO) control in cells incubated with the differentiation cocktail (Fig. 8 Diff. section) (*P* < 0.0001).

## Discussion

The first novel finding of this study, obtained by applying the MTT assay, is that both Bim and PGF_2α_EA significantly increase cell viability in 3T3-L1 preadipocytes but not in the other tested cell lines, where they were not cytotoxic either. Importantly, the observed effect was reversed by MEK-selective inhibitor PD, which was per se not cytotoxic. The MTT assay—based on cellular enzymatic activity—is widely embraced as a way of determining either drug cytotoxicity or proproliferative effects (61, 70). The potential proproliferative effect of Bim and PGF_2α_EA on preadipocytes was further confirmed by using the crystal violet assay to quantify the live cellular biomass, direct cell counting, and a BrdU incorporation-based cell proliferation ELISA that colorimetrically detects BrdU incorporation into newly synthesized DNA strands of actively proliferating cells, which all supported that both molecules induce 3T3-L1 preadipocyte proliferation. The reversal by PD was not observed in the crystal violet assay, as this dying method involves several washing steps compared to the MTT assay, which might affect its sensitivity, as also shown by the fact that the effect observed using this technique was much less noticeable than with cell counting or with the MTT assay. However, PD treatment in cell counting experiment very significantly reversed both Bim- and PGF_2α_EA-increasing effect on preadipocyte proliferation. In addition, the next experiments, which as well involved PD treatment, show that it usually reversed the effects of both molecules (see below). The overall compendium of Figs. 1 and 2 suggests the potential of Bim and PGF_2α_EA as drivers of preadipocyte multiplication and hence we next addressed the question of whether or not these molecules could affect the cell cycle (71).

Indeed, the increase in cell number was seemingly driven by an increase in cell count passing the G1-S checkpoint, as determined by flow cytometry histograms, which showed that both molecules corresponded to a greater proportion of cells within the S and G2 phases. These are the cell cycle phases that precede the mitosis phase. Although this effect was not significantly visible on separate phases (data not shown), there was a significant rise in the sum of cell percentages of these critical preparatory steps that ready the cells for cellular duplication, namely the ensemble of the S and G2 phases. Interestingly, PD treatment reversed this effect, though in a nonstatistically significant manner, yet still consistently, for both Bim and PGF_2α_EA. Importantly, the further comparison of cotreatments to vehicle control showed that PD rendered their effects no longer statistically significant. Engagingly, these results were endorsed by subsequent gene expression analyses.

In fact, the genes encoding the expression of the G1-S checkpoint regulatory CDKIs p21 (*Cdkn1a*) and p27 (*Cdkn1b*) (72) were significantly decreased in expression in response to both Bim and PGF_2α_EA treatments. Importantly, this effect, at least for p21, was reversed by selective MEK inhibition with PD for both Bim and PGF_2α_EA, which could imply a significant implication of this CDKI inhibition within the proliferative pathway we investigated herein. The lack of reversal by PD of *Cdkn1b* downregulation by Bim and PGF_2α_EA might be due to the stronger effect of the two molecules in this case. This strong effect might underscore the pertinence of altering p27 to their proliferative effects. As PD did not reverse the inhibitory effect of Bim and PGF_2α_EA on *Cdkn1b*, *Cdk4*, and *Cdk5* gene expression, we further considered checking any potential weakening effect of PD on Bim/PGF_2α_EA-lowering effect of the corresponding gene expression. For this, we further considered contrasting the two comparisons: Bim/PGF_2α_EA versus DMSO on one side and the respective lipid cotreatments with PD versus DMSO on the other side. Interestingly, PD cotreatment diminished Bim and PGF_2α_EA effect for *Cdkn1b*. This further supports a role for p27 in the studied pathway herein. The key role of p21 and p27 was further emphasized by the absence of parallel effects on other investigated genes, such as the tumor suppressor gene *Trp53* (data not shown), both with Bim and PGF_2α_EA, although this gene might still be indirectly implicated in preadipocyte proliferation (73). Indeed, the analyses of the gene expression for several CDKs globally revealed less significant results compared with CDKIs. We overall observed no significant effect on the gene expression for CDK1, 2, and 5. Furthermore, albeit there was a significant gene expression decrease for CDK4 with Bim and PGF_2α_EA, it was less significant than their effect on p21 and p27. Additionally, for *Cdk4*, the cotreatment (Bim plus PD) versus DMSO was not statistically significant compared to Bim versus DMSO which was statistically considerable. This PD-weakening effect was not observed for PGF_2α_EA. Indeed, globally, PD presented a less weakening impact on the effect on gene expression for PGF_2α_EA compared to Bim. This can be consistently illustrated by examining the gene expression patterns for p21, p27, and CDK4. Indeed, the cotreatment (PGF_2α_EA plus PD) comparison to DMSO did not reveal any reversal of PGF_2α_EA effect on *Cdk4* gene expression. The inhibitory effect of Bim and PGF_2α_EA on *Cdk4* gene expression might underlie, in part, the anti-adipogenic properties of these two molecules, given CDK4’s critical role in promoting adipogenesis (74). In sum, the effect of Bim and PGF_2α_EA was more pronounced on the gene expression of CDKIs, particularly p21 and p27, via significantly curbing both. These results agree with the flow cytometry findings and support the hypothesis that Bim and PGF_2α_EA promote cell cycle progression in 3T3-L1 preadipocytes.

Regarding the possibility of our test compounds to modulate an occurring apoptosis, DMSO (vehicle control) did not seem to induce an apoptotic phenotype (75, 76, 77). Indeed, our microscopic observations checking for apoptosis yielded photos that are similar to another study in NIH-3T3 cells (63). Additionally, 0.1% is among the lowest DMSO amounts of those commonly and safely adopted in cellular studies (78, 79). Of interest, a study on 3T3-L1 adipocytes treated with a lower DMSO dose (0.01%) showed that this dose did not affect apoptosis and that 0.1% of this solvent produced no significantly different effect from the 0.01% dose (80). When using caspase activity assay, we found that Bim and PGF_2α_EA did not present an effect on the activity of these proteases in comparison to DMSO. Importantly, the herein presented cell photos and counting further supported the finding of the proproliferative effect of both Bim and PGF_2α_EA in preadipocytes. Taken together, this indicates that they do not result in increased cell number by decreasing basal levels of apoptosis but rather by increasing cell proliferation.

Interestingly, Bim and PGF_2α_EA seem to present inducible effects. This inducibility appeared from several of our results: (I) Bim and PGF_2α_EA at concentrations lower than those that significantly induced cellular proliferation reversed, instead, the inhibitory effect of Rosi on cellular viability. (II) Accordingly, the inhibition of Rosi-induced PPARG activity was exerted at concentrations that did not affect the basal activity of this receptor. (III) Finally, and perhaps most importantly, we observed a clearly more pronounced increase of cellular viability by Bim and PGF_2α_EA during preadipocyte differentiation. We showed previously that during the first two days of adipogenesis, Bim and PGF_2α_EA reduce the expression of the key adipogenic genes *Pparg* and *Cebpa* (21). This stage of early adipogenesis is reported to be characterized by the preparatory mitotic clonal expansion that multiplies 3T3-L1 preadipocytes a few times after their postconfluence growth arrest, although this is not consensually established (81, 82). It is interesting that while anti-adipogenic, Bim and PGF_2α_EA also increased cell viability in postconfluent Diff. cells. Such binary functional effect is rarely reported in the literature, which could emphasize the distinguished role of these molecules (83, 84, 85). An example of this dual impact could be the biomolecule Rev-erbα, which is both mitotic and inhibitor of *Pparg* expression in these cells (86). Boosting cell proliferation during early adipogenesis, by Bim and PGF_2α_EA, could be of high importance, as while anti-adipogenic, Bim and PGF_2α_EA might also actively multiply preadipocytes to guard a pool of these cells available for WAT formation that could be needed by adipogenic signals. Interestingly, this effect of Bim and PGF_2α_EA was also observed in Undiff. cells, which supports the role of these molecules in guarding the preadipocyte reserve. Accordingly, this effect was stronger when this pool was challenged by differentiation triggering. Globally, Bim and PGF_2α_EA effects tend to maintain the preadipocyte pool, through enhancing its cellular multiplication and inhibiting its differentiation. This reserve of adipocyte precursors might promote the healthy alternative for fat storage through fostering hyperplasia over unhealthy hypertrophy (17), thus suggesting that endogenous PGF_2α_EA plays a role in maintaining healthier WAT plasticity.

It is noteworthy that PGF_2α_EA is a potential pivotal regulator of WAT fat-storing dynamism, as it is an anti-adipogenic metabolite of the pro-adipogenic PPARG ligand, that is, AEA (30), and it is suggested thus as a feedback controller of adipogenesis process (21, 53). Indeed, PGF_2α_EA levels are lower in high fat diet-fed mice WAT, when the adipose tissue needs expanding, suggesting that its levels are dynamically regulated in the presence of signals stimulating WAT expansion (21). This might further support its role in supporting healthy fat storage, which is lost in morbid obesity (17), by ensuring a healthy pool of adipocyte precursors, ready to differentiate when needed. Importantly, Bim and PGF_2α_EA effects seem to be rather safe, in terms of being reversible, as glaucoma patients regain their ocular fat pads with Bim treatment cessation (47). It is further interesting that PGF_2α_EA presented no proliferative effect in the other tested cell lines herein, and it also exhibited a targeted effect as it did not equally affect all the analyzed genes controlling the cell cycle while exerting no impact on the tumor-suppressor gene *Trp53*. These observations point to a selective and specific, rather than uncontrolled and ubiquitous, action of PGF_2α_EA and further strengthen its position as a promising preadipocyte regulating mediator, thus stimulating more research within the field of obesity. Captivatingly, the findings we are presenting herein with Bim and PGF_2α_EA suggest mutually corroborative patterns for the two molecules. Also important is the present observation that the effects of Bim and PGF_2α_EA seem to be both mediated by the same mechanism involving MAPKK, as shown for instance by the PPARG activity-curbing effect of both molecules being significantly reversed by PD. Compellingly, MAPK signaling is not only the most established driver of cellular proliferation (54) but also a well-documented inhibitory pathway of the activity of PPARG through inducing its phosphorylation (87, 88, 89). This goes in line with our results on cell cycle promotion and PPARG activity inhibition.

This work should foster new studies on Bim, whose use has been so far limited to ophthalmic eye drops, whereas explorations about its effect on the WAT have been relatively dropwise, though potentially impactful (21). Indeed, a recent important study on Bim cutaneous administration in rats showed its ability to reduce body weight gain and to enhance satiety (53). Our results might propose supporting explanations to these recent findings. However, our data need to be first confirmed in primary cultures of murine preadipocytes and, particularly, human WAT in order to confirm that these previous observations with Bim are explained by a proproliferative action on preadipocytes. Our work could as well incentivize basing research hypotheses on empirical observations besides literature-based findings (90). For example, observing cell pellet size was a cue for this paper hypothesis. Interestingly, this observation can originate several more research paths, such as exploring the cellular morphology and size, for the scientific community.

## Conclusions

**Fig. 9:**
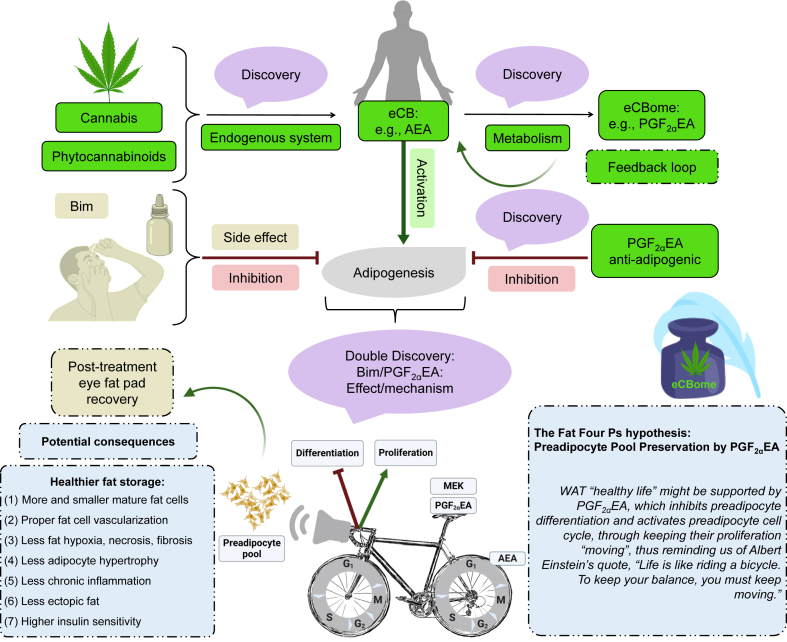
Prostaglandin F_2α_ ethanolamide (PGF_2α_EA) and bimatoprost (Bim) potential effects. A chain of discoveries resulted from research on the endocannabinoid system (eCBS). The discovery that certain *Cannabis sativa* phytocannabinoids activate the body cannabinoid receptors CB1 and CB2 had directed researchers to discover the eCBS and the endocannabinoids (eCBs), as AEA (93, 94). PGF_2α_EA is an eCB (AEA) bioactive metabolite, which belongs to the wider lipid signaling network—the endocannabinoidome (eCBome) (34). This paper adds a new ring to the eCBome cognizance chain to ring a bell about its noteworthy roles. We showed that the chief eCB mediator AEA metabolite PGF_2α_EA induces preadipocyte proliferation and inhibits PPARG activity, in a mitogen-activated protein kinase kinase (MAPKK)-dependent fashion, which complements our previous discovery of its anti-adipogenic effect (21). We suggest that PGF_2α_EA might favor healthier fat storage and white fat plasticity, to outbalance obesity complications driver—hypertrophy (17). We parallelly showed that its pharmaceutical analog Bim presented similar effects, which might provide support in explaining the quick recovery of ocular fat pads with drug cessation. It is time o’clock to recognize and update our pathophysiology books to include the eCBome (34), especially to further revive obesity research (23, 24, 95, 96, 97, 98). We further emphasize the potential power of the research virtuous circle; clinical observation-research-discovery-clinical application, both within the same discipline and also beyond it to link relatively unrelated disciplines, such as ophthalmology and obesity research (53, 99), to guide towards more efficient multidisciplinary and interdisciplinary discoveries. This figure and the graphical abstract were created with BioRender.com and with MindtheGraph.com. Fig. 9 ink bottle icon was made by Luvdat from www.flaticon.com. AEA, *N*-arachidonoyl-ethanolamine; PPARG, peroxisome proliferator-activated receptor gamma.

Our report provides novel insights to overcome the current knowledge hiatus about preadipocyte physiology, upgrades our knowledge about the eCBome, and offers findings of potential pharmaceutical value for obesity and ophthalmology research for the potential development of new therapies based on this sophisticated signaling system. Bim is already widely prescribed for treating glaucoma, and its side effect of increasing eyelashes size widened its use as an anti-hypotrichosis cosmetic under the name Latisse (90, 91). Hence, further research on its preadipocyte proliferative and anti-adipogenic effects might also prompt its potential use as a therapy towards “healthy” obesity and against the ectopic fat accumulation that normally accompanies morbid obesity. Additionally, our present work might point, for the first time, to PGF_2α_EA as a keeper of WAT preadipocyte pool, through MAPKK-dependent inhibition of its transformation and acceleration of its multiplication cycle. We would like to name this hypothesis the “Fat Four Ps hypothesis”, that is, Preadipocyte Pool Preservation by PGF_2α_EA. It might be paraphrased with PGF_2α_EA role to maintain WAT “healthy life” by activating the preadipocyte cell cycle, through keeping the proliferation “moving”, thus reminding us of Albert Einstein’s quote, “Life is like riding a bicycle. To keep your balance, you must keep moving.” (92), as schematized in the graphical conclusion depicted in Fig. 9.

## Data Availability

All data are contained within the manuscript.

## Conflicts of interest

The authors declare that they have no conflicts of interest with the contents of this article.
